# Sea‐level rise, habitat loss, and potential extirpation of a salt marsh specialist bird in urbanized landscapes

**DOI:** 10.1002/ece3.4196

**Published:** 2018-07-22

**Authors:** Jordan A. Rosencranz, Karen M. Thorne, Kevin J. Buffington, John Y. Takekawa, Ryan F. Hechinger, Tara E. Stewart, Richard F. Ambrose, Glen M. MacDonald, Mark A. Holmgren, Jeff A. Crooks, Robert T. Patton, Kevin D. Lafferty

**Affiliations:** ^1^ Western Ecological Research Center U.S. Geological Survey San Francisco Bay Estuary Field Station Vallejo California; ^2^ Institute of the Environment and Sustainability University of California Los Angeles California; ^3^ Suisun Resource Conservation District Suisun City California; ^4^ Marine Science Institute University of California Santa Barbara California; ^5^ Marine Biology Research Division Scripps Institution of Oceanography University of California, San Diego La Jolla California; ^6^ Department of Ecology, Evolution and Marine Biology University of California Santa Barbara California; ^7^ Program in Ecology, Evolution, and Conservation Biology University of Illinois Urbana‐Champaign Illinois; ^8^ Department of Environmental Health Sciences University of California Los Angeles California; ^9^ Department of Geography University of California Los Angeles California; ^10^ Cheadle Center for Biodiversity and Ecological Restoration University of California Santa Barbara California; ^11^ Tijuana River National Estuarine Research Reserve Imperial Beach California; ^12^ Avian Research Associates Coronado California; ^13^ Western Ecological Research Center c/o Marine Science Institute U.S. Geological Survey University of California Santa Barbara California

**Keywords:** Belding's savannah sparrow, California, conservation, dynamic salt marsh accretion model, sea‐level rise, species distribution model

## Abstract

Sea‐level rise (SLR) impacts on intertidal habitat depend on coastal topology, accretion, and constraints from surrounding development. Such habitat changes might affect species like Belding's savannah sparrows (*Passerculus sandwichensis beldingi*; BSSP), which live in high‐elevation salt marsh in the Southern California Bight. To predict how BSSP habitat might change under various SLR scenarios, we first constructed a suitability model by matching bird observations with elevation. We then mapped current BSSP breeding and foraging habitat at six estuarine sites by applying the elevation‐suitability model to digital elevation models. To estimate changes in digital elevation models under different SLR scenarios, we used a site‐specific, one‐dimensional elevation model (wetland accretion rate model of ecosystem resilience). We then applied our elevation‐suitability model to the projected digital elevation models. The resulting maps suggest that suitable breeding and foraging habitat could decline as increased inundation converts middle‐ and high‐elevation suitable habitat to mudflat and subtidal zones. As a result, the highest SLR scenario predicted that no suitable breeding or foraging habitat would remain at any site by 2100 and 2110. Removing development constraints to facilitate landward migration of high salt marsh, or redistributing dredge spoils to replace submerged habitat, might create future high salt marsh habitat, thereby reducing extirpation risk for BSSP in southern California.

## 1 INTRODUCTION

Salt marshes shift their distributions in response to sea‐level rise (SLR) through vertical accretion, landward inundation, and retreat to formerly dryland sites (Donnelly & Bertness, 2001). Anticipated future rapid rates of SLR could obviate the benefits of accretion, and coastal development will, in many cases, prevent inland retreat (Roman, 2017). Along the Pacific Coast, recent modeling efforts have predicted a complete loss of coastal salt marshes in California (Thorne et al., 2018). This is of particular concern in areas such as southern California (USA), where small, “urban” salt marshes are hotspots and refugia for sensitive endemic species (Zedler, 1982), including the state endangered Belding's savannah sparrow (*Passerculus sandwichensis beldingi*; BSSP). Here, we examine how BSSP habitat may respond to SLR.

Estuarine sparrows have small home ranges and narrow elevation niches. Any benefits of sediment contributions could be obscured, reducing niche availability, and potentially geographic ranges, for those species dependent upon estuarine margins. For instance, salt marsh sparrows (*Ammodramus caudacutus*) on the USA east coast declined by 9% annually, from 1998 to 2012, primarily due to reductions in habitat availability. Such losses could be compounded by SLR, with studies predicting extirpation by 2035 (Field et al., 2017) or 2050 (Correll et al., 2016). Furthermore, salt marsh habitat for seaside sparrow (*Ammodramus maritimus*) populations in Georgia, USA, could contract by 81% by 2100 (Hunter, Nibbelink, & Cooper, 2016). Similarly, in the San Francisco Bay Estuary (SFBE), the tidal marsh song sparrow (*Melospiza melodia*) could be vulnerable as high‐elevation habitat becomes low elevation habitat (Veloz et al., 2013). However, Kirwan, Temmerman, Skeehan, Guntenspergen, and Fagherazzi (2016) suggest that past models based on constant accretion rates do not incorporate the self‐adaptive capacity of salt marshes, thereby over‐estimating habitat loss. Future projections of habitat response to SLR that account for the dominant processes dictating tidal marsh elevation (Morris, Sundareshwar, Nietch, Kjerfve, & Cahoon, 2002; Schile et al., 2014; Swanson et al., 2014; Thorne et al., 2018), species distribution modeling, and population size projections should provide more robust projections of suitable habitat.

Similar to Atlantic Coast and SFBE salt marsh sparrows, BSSP is a nonmigratory salt marsh specialist with a narrow geographic range from Goleta Slough, California, USA, to Bahia San Quintin, Baja California, Mexico. BSSP breeding depends on middle‐ to high‐elevation marsh habitats dominated by pickleweed (*Sarcocornia pacifica*) and salt grass (*Distichlis spicata*) (Bradley, 1973, 1994; Grinnell & Miller, 1944; Powell, 1993, 2006). Inundation limits BSSP's lower elevation niche, whereas territorial song sparrows (*M. melodia*) can displace BSSP from the upland transition zone (Zembal, Hoffman, & Patton, 2015). Furthermore, studies of BSSP song dialects suggest that individuals do not often disperse between sites (Bradley, 1994; Burnell, 1996), subjecting them to local extirpation. In 1988, BSSP occupied only 2,150 ha of salt marsh vegetation, salt flats, and small tidal channels among 27–30 sites on the Southern California Bight (SCB) (Zembal, Kramer, Bransfield, & Gilbert, 1988), varying in area from <1 ha to 620 ha (Powell, 2006). The 2015 regional population estimate found an increase of 11.3% from counts in 2010 (Zembal et al., 2015), perhaps due to greater nesting success and survival in a warmer and drier period. How SLR affects this apparent recovery trajectory could affect plans for delisting.

Salt marsh habitats lie within the intertidal zone and rely on a balance between accretion and erosion, as well as uplift and subsidence to maintain elevations with SLR. Salt marshes can trap mineral sediment and accumulate organic matter to maintain their position with rising seas (Kirwan et al., 2016), and they might migrate inland as upland habitats recede (Raabe & Stumpf, 2016). However, coastal development in the SCB acts as a backstop to transgression and likely reduces sediment available for accretion (Callaway & Zedler, 2004; Thorne et al., 2018). Nonetheless, diverse land uses within each salt marsh catchment lead to variable accretion rates within and across salt marshes in the SCB (Callaway, Borgnis, Turner, & Milan, 2012; Day et al., 1999; Thorne et al., 2018). The uncertainty created by the range in accretion rates led us to create vulnerability scenarios for individual salt marshes using site‐specific data, as well as build upon recent Pacific Coast research that predicts salt marsh eradication within 100 years under high rates of SLR (Thorne et al., 2018).

To project future salt marsh elevations at sites that are important to BSSP, we calibrated the wetland accretion rate model of ecosystem resilience (WARMER). WARMER is a one‐dimensional soil cohort model that projects future salt marsh elevation based on (a) the dynamic relationship between organic matter accumulation and elevation, (b) the nonlinear relationship between inorganic matter accumulation and elevation, and (c) temporally variable SLR (Swanson et al., 2014; Thorne et al., 2018). Unlike the regional and temporally constant accretion rate used to calibrate wetland models for other sites in other studies (Kirwan et al., 2016), we used in‐situ historical sediment accumulation rates to dynamically project organic and mineral accretion for each salt marsh.

To indicate how BSSP geographic ranges could shift over time, we coupled the WARMER projections with a species distribution model (Maxent; Phillips, et al. 2006; Phillips et al., 2018) in R (R Core Team, 2018). Maxent models habitat suitability indices (Barbosa & Schneck, 2015) from presence‐only data (Merow, Smith, & Silander, 2013). Maxent can also calculate objective threshold values (e.g., 10‐percentile threshold) to generate species distribution maps (Liu, Berry, Dawson, & Pearson, 2005; Wakie, Evangelista, Jarnevich, & Laituri, 2014).

## 2 MATERIALS AND METHODS

To assess the vulnerability of BSSP habitat to SLR, we (a) collected baseline habitat information, (b) estimated salt marsh area and elevation gains or losses with SLR using a dynamic one‐dimensional elevation model, (c) determined current habitat suitability, and (d) projected habitat suitability under three plausible SLR scenarios. Fine‐scale, site‐specific data were leveraged to answer these research questions at 5‐m horizontal resolution across six study sites in the SCB.

### 2.1 Habitat modeling with SLR

#### 2.1.1 Study sites

**Figure 1:**
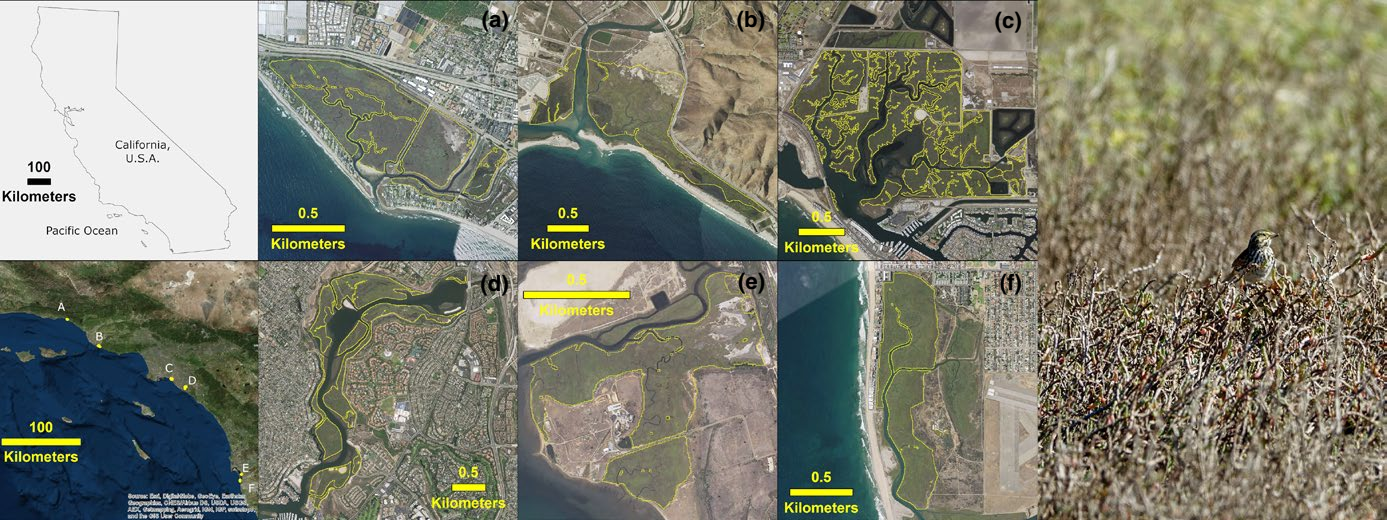
Photograph of Belding's Savannah Sparrow (*Passerculus sandwichensis beldingi*) at Mugu during a 2018 survey, and polygons (yellow boundaries) of modeled salt marshes [Carpinteria (a), Mugu (b), Seal Beach (c), Newport (d), Sweetwater (e), and Tijuana (f)]

We modeled habitat suitability for the six fully tidal salt marshes (Carpinteria Salt Marsh Reserve [Carpinteria], Mugu Lagoon within Naval Base Ventura County [Mugu], Seal Beach National Wildlife Refuge [Seal Beach], Upper Newport Bay [Newport], Sweetwater marsh within the San Diego Bay National Wildlife Refuge (Sweetwater], and the north arm of the Tijuana Slough National Wildlife Refuge [Tijuana]) (Figure 1) where BSSP breed now and enough data were available to parameterize WARMER (see Elgin, 2012 and Thorne et al., 2014, 2016).

#### 2.1.2 Salt marsh topography

For evaluating salt marsh vulnerabilities, salt marsh elevations were defined relative to the local tide datum. Swanson et al. (2014) define z* as a unit free “elevation relative to the tidal range of the site,” which is calculated as:

(1) $$z^{*}=\left(z\text{‐}\text{Mean}\;\text{Sea}\;\text{Level}\right)/\left(\text{Mean}\;\text{Higher}\;\text{High}\;\text{Water}-\text{Mean}\;\text{Sea}\;\text{Level}\right)$$

By definition, *z* = the absolute elevation relative to North American Vertical Datum 1988 (NAVD88). Because z* = 0 when *z* = mean sea level (MSL), and z* = 1.0 when *z* = MHHW for all sites, we were able to compare vulnerabilities across sites.

Site‐specific current topography data were obtained for SCB salt marshes at all sites (Sadro, Gastil‐Buhl, & Melack, 2007; Thorne et al., 2014, 2016). Elevation data were measured using Leica RX1200 Real Time Kinematic (RTK) Global Positioning System (GPS) rover (±1 cm, *x*,* y*; ±2 cm *z* accuracy; Leica Geosystems Inc., Norcross, Georgia; http://www.leica-geosystems.com) at all sites with the exception of Carpinteria, where the researchers used Topcon GTS‐213 for ground surveys (details in Sadro et al., 2007). Thorne et al. (2014) describes the RTK surveys at Sweetwater, and Thorne et al. (2016) describes the survey methods for the remaining sites. In ground surveys at all six sites, a small base plate helped prevent the survey instrument from sinking into the mud. Modeled areas included vegetated zones within each salt marsh, excluded large tidal creeks, levees, adjacent bluffs, and roads, and assumed levees or human infrastructure such as roads and houses prevented transgression into upland areas.

Two methods helped enhance and expand current digital elevation models (DEMs) to include natural salt marsh habitat. First, elevations at Carpinteria were obtained from a previously published DEM that corrected for dominant vegetation interference (Sadro et al., 2007). At Tijuana South, where we conducted data training, we obtained a submeter pixel‐sized DEM from 2014 (San Diego CA 2014 LiDAR USGS Contract: G10PC00026). At Tijuana South, as well as the other five sites, the LEAN (Lidar Elevation Adjustment using Normalized Difference Vegetation Index, or NDVI) correction technique (Buffington, Dugger, Thorne, & Takekawa, 2016) was used to adjust bare earth 1 m Lidar (California Coastal Conservancy Coastal Lidar Project: 2009–2011; https://coast.noaa.gov/htdata/raster2/elevation/California_Lidar_DEM_2009_1131/) for the positive bias due to dense vegetation. LEAN uses the Normalized Difference Vegetation Index (NDVI) from existing 1 m National Agricultural Inventory Program imagery to model the vertical error in 1 m lidar. The LEAN model was calibrated with the site‐specific RTK elevation survey and NDVI data, and a correction was applied to the bare earth DEM of each site. Lidar site boundaries were similarly defined by natural vegetation breaks.

#### 2.1.3 Wetland Accretion Rate Model of Ecosystem Resilience

WARMER is a dynamic, one‐dimensional elevation model that incorporates the self‐adaptive capacity of salt marshes to respond to SLR based on site‐specific inundation, sedimentation, climate, and vegetation characteristics (Callaway, Nyman, & DeLaune, 1996; Swanson et al., 2014). Based on a cesium‐137 analysis of a soil core from Carpinteria via Elgin (2012), existing soil core parameters from Sweetwater via Thorne et al. (2014) and soil core parameters from the remaining sites via Thorne et al. (2016), we used the full WARMER model, and all associated inorganic sediment and organic matter functions (Morris et al., 2002; Swanson et al., 2014), to project California salt marsh habitat based on three potential SLR scenarios (low [+0.44], moderate [0.93], and high [1.66 m/100 years]) predicted for California coastal regions south of Cape Mendocino (National Resource Council 2012). Inputs to WARMER include site‐specific belowground soil properties, aboveground vegetation, inundation and sediment characteristics, including relative SLR, above ground productivity, and sediment input (Table 1). WARMER was run at a range of initial elevations, and projected changes in elevation were interpolated onto the DEM of each site.

**Table 1:** Parameters used to run Wetland Accretion Rate Model of Ecosystem Resilience at Carpinteria (CA), Mugu (MU), Seal Beach (SE), Newport (NE), Sweetwater (SW), and Tijuana (TI)

Parameter	CA^a^	MU^b^	SE^b^	NE^b^	SWb,c	TI^b^
Area (ha)	65	138	200	151	43	58
Sediment accumulation rate ([g/cm^2^]/year)	0.9	1.4	0.3	0.2	0.2	0.2
Elevation of peak biomass (cm, MSL)	108.6	87.9	92.0	82.2	73.2	56.0
Minimum elevation of vegetation (cm, MSL)	−1.4	30.9	2.0	−0.8	11.2	−34.0
Maximum aboveground organic accumulation ([g/cm^2^]/year)	0.04	0.17	0.03	0.01	0.05	0.02
Root‐to‐shoot ratio	0.46	0.46	0.46	0.46	0.46	0.46
Porosity at the surface (percent)	88	60	87	86	87	60
Porosity at depth (percent)	59	41	38	45	74	39
Refractory carbon (percent)	20.6	5.9	8.9	27.1	7.0	28.0
Maximum astronomical tide (cm, MSL)	135	118	157	130	136	150
Historical SLR (mm/year)	1.1	2.3	2.2	2.1	2.1	2.2
Organic matter density (g/cm^3^)	1.1	1.1	1.1	1.1	1.1	1.1
Mineral density (g/cm^3^)	2.6	2.6	2.6	2.6	2.6	2.6

^a^Sediment core parameters from Elgin (2012). ^b^Sediment core parameters from Thorne et al. (2016). ^c^Sediment core parameters from Thorne et al. (2014).

### 2.2 Belding's savannah sparrow occurrence

BSSP transect surveys occurred at Carpinteria in 2012–2013 and Mugu in 2018. Additional breeding season survey data were compiled from surveys conducted on 14, 27, 28, and 29 April 2004 and 6 May 2005 at Border Field State Park, which is adjacent to the Tijuana study site. Methods are described in Rick Engineering Company (2008), but the protocol followed that established for the 5‐year statewide breeding survey of the species by California Department of Fish and Wildlife (Zembal et al., 2015). In summary, biologists walked transects and sightings within 100 feet of the transects were recorded on field maps and later georeferenced over an aerial photograph. Breeding habitat was indicated by several categories of BSSP behaviors such as singing (s) or scolding, perching together of mates, including feeding of young, nest building, and aerial chases between two territory holders. Posted males or foraging birds were not included in the breeding habitat or breeding period model inputs. All areas surveyed were accessible on foot.

The Carpinteria transect surveys occurred on two consecutive days monthly from January 2012 to March 2013, spanning high and low tides (Lafferty, Stewart, & Hechinger, 2017). A survey occurred on a single day at Mugu Lagoon in February 2018 using a similar transect survey strategy. The walking transect was designed to sample the breeding and foraging habitat exhaustively. All observed BSSP were recorded to the nearest 10 m on hard copy maps, and points were later digitized in ArcGIS. BSSP, especially nonsinging males, are secretive and difficult to detect at distance (Powell, 2006). Distance sampling was performed post hoc, so every digitized sparrow location was assigned a distance to the transect. In this study, counts declined by half when 30 m from an observer so that we had high confidence in detections within 10 m of a transect.

The compiled data at Border Field State Park followed the 5‐year statewide breeding survey protocol (Zembal et al., 2015), where occurrences within breeding territories were indicated by behaviors such as singing. Because Tijuana was the only site where BSSP observations were coded as breeding and at a suitable scale to put into our model, we estimated the period breeding for Carpinteria based on breeding occurrences at Devereux Slough, Carpinteria, Santa Ynez River Estuary, and Goleta Slough (Holmgren, & O’Loghlen 2018). Breeding periods included egg lay (5 days), incubation (13 days), nestling (8 days), postnestling and parental care (7–14 days). The two earliest breeding records used to establish the onset of breeding came from Devereux Slough.

#### 2.2.1 Maxent modeling

We developed two simple Maxent models for BSSP breeding and foraging habitat suitability using representative BSSP occurrence observations and site‐specific elevation data. Although other habitat aspects might drive BSSP density, we limited the environmental layer to elevation because this is the primary variable subject to change under SLR and covaries with numerous other environmental variables likely to be important for BSSP (e.g., exposure time, plant composition, salinity). We trained the Maxent models with data from Carpinteria, Mugu, and Tijuana because those were the only sites where we had occurrence data at a scale relevant to our modeling approach. At Carpinteria and Mugu, observations were excluded if they fell outside the predetermined marsh extent or beyond 10 m of a survey transect. We assumed that breeding territories were exhaustively mapped within the Borderfield State Park because territorial behaviors are more conspicuous and could be detected at a greater distance.

We assumed all BSSP observations represented suitable foraging habitat and developed a subset of observations that represented breeding habitat based on seasonal and behavioral cues. At Carpinteria and Mugu, observations were coded as breeding if they were within the March 11 to August 19 breeding period. Individuals exhibiting breeding behavior at Borderfield State Park were included in the breeding dataset. In the breeding dataset, we excluded points from model training that were below z* = 0 because it was unlikely that the species would nest or exhibit breeding behavior in that zone (i.e., birds were likely using that mudflat habitat for foraging). Across the three sites, we had 1,595 BSSP observations that we assigned to the foraging dataset, with a subset of 571 observations assigned to the breeding period, which likely included some foraging observations; and a subset of 229 observations assigned breeding habitat, which were only included in the breeding behavior dataset. Because of the small size of the study sites, the number of background points was density‐dependent (1,000 points per km^2^); other Maxent parameters were kept as default. We projected current and future BSSP foraging and breeding habitat suitability in decadal increments to 2110 and under the three SLR scenarios using the WARMER elevation projections.

### 2.3 Estimating habitat change

To simplify analyses and presentation, we defined habitat as suitable if the elevation was within the 90th percentile of the occurrence probability distribution, a standard often used to delineate conservation priority areas (Fourcade, Engler, Rödder, & Secondi, 2014; McFarland et al., 2013; Wakie et al., 2014). We used the 10‐percentile training presence threshold to recode the continuous Maxent output (probability of occurrence) to suitable or unsuitable habitat and calculated the area to determine percent suitable habitat change in the representative fully tidal basins in the SCB. For the suitable breeding habitat (Borderfield), breeding period (Carpinteria and Mugu), and foraging habitat models (all three sites), the 10‐percentile training presence threshold values were 0.344, 0.365, and 0.421, which represents the probability of occurrence above which suitable habitat occurs.

## 3 RESULTS

### 3.1 Current distribution

**Figure 2:**
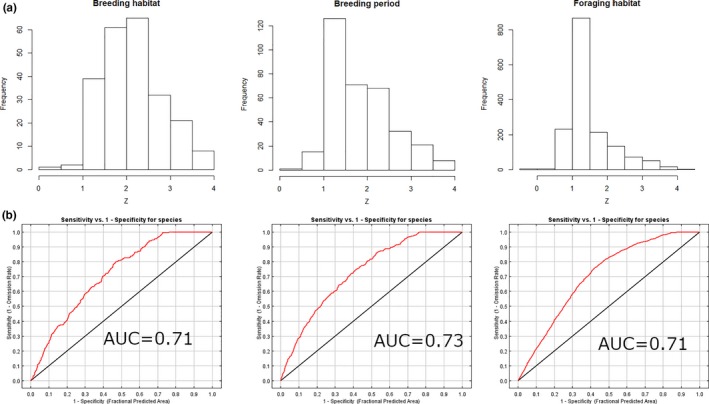
Results from (a) histogram showing relationship of Belding's Savannah Sparrow (*Passerculus sandwichensis beldingi*) breeding habitat suitability to relative elevation (z*) for three modeled habitat types, and (b) Maxent receiver operator curves for each model

**Figure 3:**
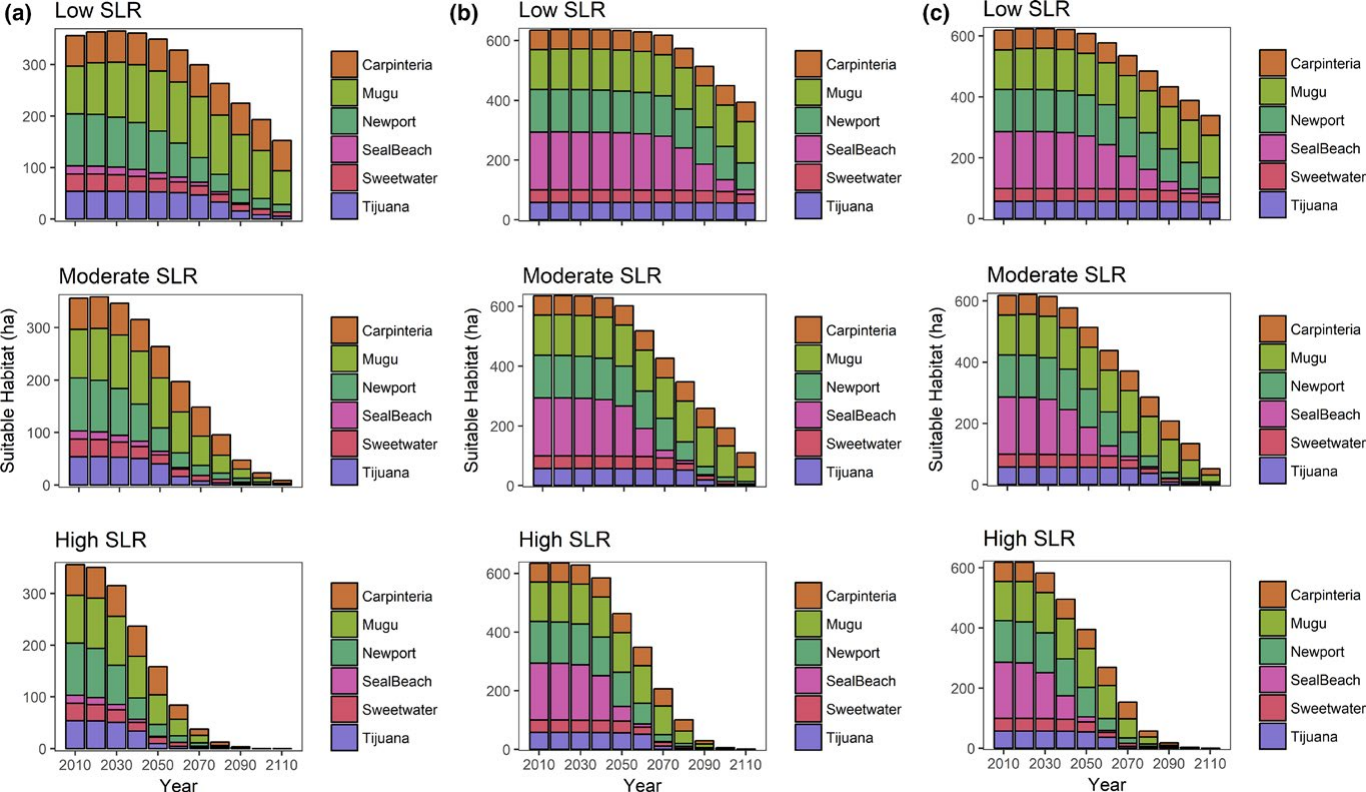
Scenarios showing change in suitable (a) breeding habitat, (b) breeding period habitat, and (c) foraging habitat across full tidal salt marshes over three plausible sea‐level rise scenarios in the Southern California Bight

**Figure 4:**
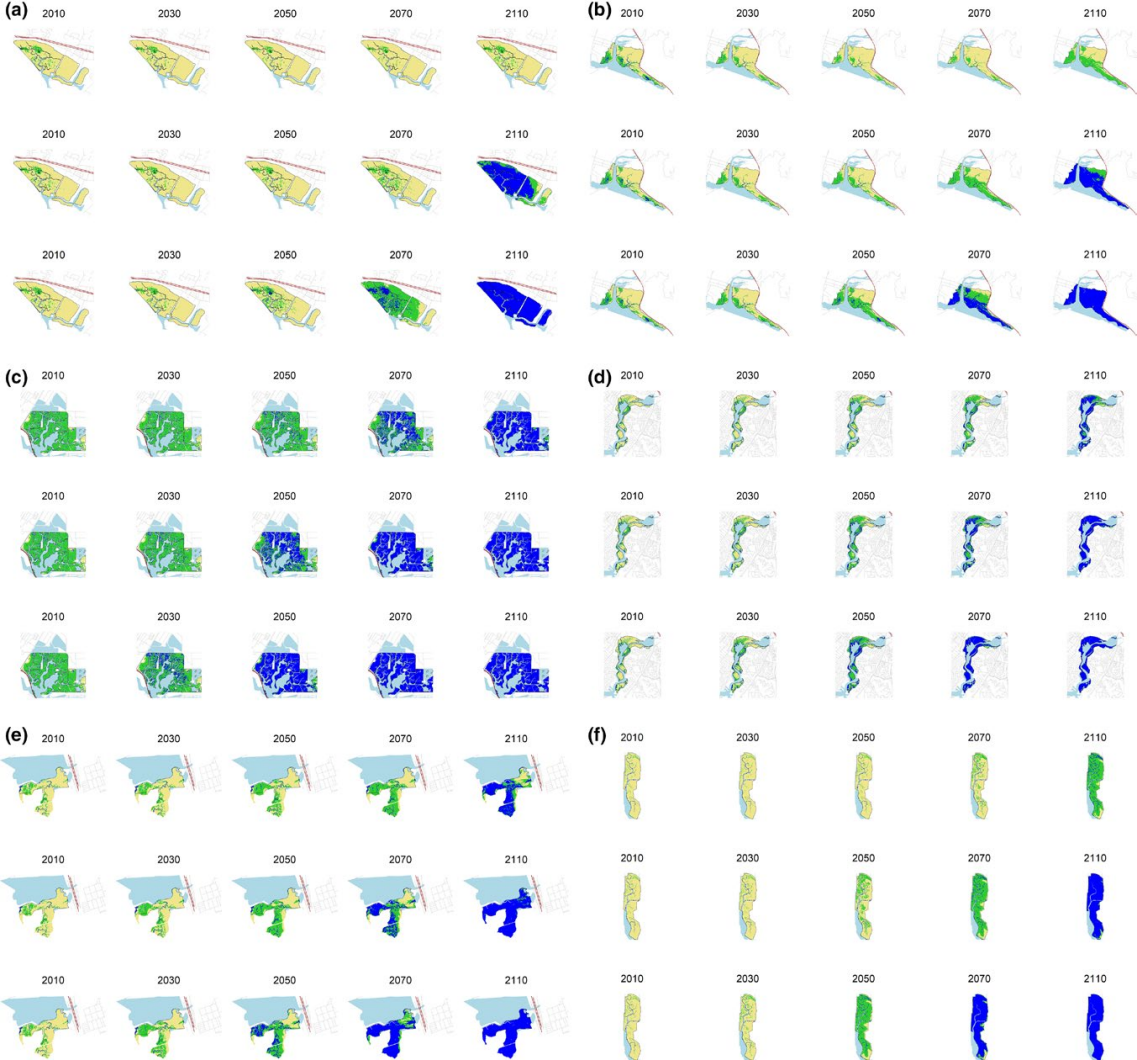
Scenarios showing how suitable breeding habitat area (breeding = beige, foraging = green, and blue = submerged) could change overtime at (a) Carpinteria, (b) Mugu, (c) Seal Beach, (d) Newport, (e) Sweetwater, and (f) Tijuana under low (0.44 m/100 year; top row), moderate (0.93 m/100 year; middle row), and high (1.66 m/100 year; bottom row) sea‐level rise projections

Suitable breeding habitat occurred in the middle, high, and upland transition zones of the saltmarsh, while foraging habitat was more extensive and variable across sites. This was defined by Maxent modeling, but quantiles explain the elevation differences. For example, the 90% quantile of z* (a dimensionless elevation value; Swanson et al., 2014) for breeding habitat, breeding period, and foraging habitat were 3.9, 2.9, and 2.4, respectively (Table 2). The response to elevation was better than the random species distribution model (Figure 2), and results of the Maxent modeling indicate that 99% of the Carpinteria salt marsh was suitable foraging habitat and 91% (59 ha) was suitable breeding habitat (Figures 3 and 4). Approximately 67% (93 ha) of Mugu was suitable breeding habitat, and 94% (130 ha) was suitable for foraging. Conversely, although 93% (186 ha) of Seal Beach was suitable foraging habitat, only 8% (16 ha) was suitable breeding habitat (Figure 3). At Newport, 90% (138 ha) of the salt marsh was suitable for foraging and 67% (101 ha) was suitable as breeding habitat. At Sweetwater, most (97%; 42 ha) of Sweetwater was suitable for foraging habitat, and 78% (34 ha) was suitable for breeding. Finally, at Tijuana, 91% (53 ha) of salt marsh was suitable breeding habitat, while 90% (53 ha) was suitable foraging habitat (Figures 3 and 4). Thus, within and across fully tidal salt marshes, there was a mosaic of suitable breeding and foraging habitat. However, these relative percentages changed with projected SLR.

**Table 2:** Quantiles of z* for each modeled habitat type. Breeding habitat was defined by occurrences that were coded as breeding behavior, and breeding period habitat was defined by occurrences that fell within a measured breeding period (March 11–August 19)

Quantiles	0%	10%	20%	30%	50%	70%	90%	100%
Breeding habitat (z*)	0.40	1.24	1.54	1.76	2.03	2.45	3.11	3.84
Breeding period (z*)	0.40	1.07	1.16	1.23	1.68	2.14	2.93	3.84
Foraging (z*)	−0.12	0.94	1.03	1.08	1.20	1.54	2.44	4.25

### 3.2 Low rates of SLR (0.44 m/100 year)

The distribution of habitat was predicted to shift extensively under a low rate of SLR. However, Carpinteria was an exception. Under this scenario, 99% of the salt marsh was predicted to be suitable foraging habitat by 2110, and Carpinteria was expected to have no net loss of suitable breeding habitat (Figures 3 and 4). Mugu also was projected to gain suitable foraging habitat under this scenario. However, Mugu was predicted to lose breeding habitat so that only 47% (65 ha) of the salt marsh could be suitable by 2110 (Figures 3 and 4). At Seal Beach, foraging habitat was expected to be reduced to 5% (9 ha), and breeding habitat was expected to be eliminated by 2110 (Figures 3 and 4). Newport foraging habitat was predicted to be reduced to less than 36% (54 ha) of the total salt marsh area, while breeding habitat was expected to be reduced to less than 10% (15 ha) of the total salt marsh area (Figures 3 and 4). Sweetwater could consist of 44% (19 ha) of suitable foraging habitat and 19% (8 ha) of suitable breeding habitat by 2110. Within 100 years, nearly 90% (53 ha) of Tijuana could be characterized as foraging habitat, while only 9% (5 ha) of breeding habitat is expected to be left. Therefore, substantial breeding habitat area across the SCB could be converted to foraging habitat by 2110 under a low SLR.

### 3.3 Moderate rates of SLR (0.93 m/100 year)

Large swaths of breeding and foraging habitat were predicted to be submerged under a moderate rate of SLR, with local extirpations occurring in some salt marshes. Under this scenario, 32% (21 ha) of Carpinteria was expected to be suitable foraging habitat by 2110, and Carpinteria was expected to be left with 9% (5.6 ha) of breeding habitat (Figures 3 and 4). At Mugu, suitable foraging habitat was predicted to be reduced to 16% (22 ha) of the total area under this scenario, and less than 1 ha of the salt marsh was predicted to be suitable breeding habitat by 2110 (Figures 3 and 4). At Seal Beach, foraging and breeding habitat were expected to be reduced to less than 1 ha by 2110 and 2070 (Figures 3 and 4). Further south, Newport foraging habitat was expected to be reduced to 4% (6 ha) of the initial salt marsh area, while breeding habitat could be reduced to 1% (2 ha) of the initial salt marsh area (Figures 3 and 4). Sweetwater was expected to have 4% (2 ha) of suitable foraging habitat left and to consist of less than 1 ha of suitable breeding habitat by 2100. A similar pattern was observed at Tijuana, where 4% (2 ha) was expected to be foraging habitat by 2110, and 2% (1 ha) was expected to be breeding habitat by 2100. Thus, under a moderate SLR scenario, local extirpations of BSSP could be expected.

### 3.4 High rates of SLR (1.66 m/100 year)

This scenario showed complete submergence of foraging and breeding habitat at all six sites by 2110. Under this scenario, 5% (3 ha) of Carpinteria was predicted to be suitable foraging and suitable breeding habitat by 2100, and no BSSP habitat was expected to be left by 2110. At Mugu, suitable foraging habitat was expected to be reduced to 1% (2 ha) of the total area by 2090 under this scenario, and less than 1 ha of the salt marsh was predicted to be suitable breeding habitat by 2100 (Figures 3 and 4). At Seal Beach, foraging and breeding habitat were eliminated by 2070 and 2060 (Figures 3 and 4). Only 2% (3 ha) of Newport foraging habitat was expected to be left by 2090, while breeding habitat was predicted to be reduced to 4% (7 ha) of the initial salt marsh area by 2070 (Figures 3 and 4). At Sweetwater, less than 1 ha of foraging and breeding habitat was expected to be left by 2090 and 2080. At Tijuana, where less than 1 ha was predicted to be foraging habitat by 2100, and less than 1 ha was expected to be breeding habitat by 2080. All suitable habitats were expected to be submerged in Tijuana by 2110. Thus, BSSP is expected to have no suitable habitat left under a high SLR scenario by 2110.

### 3.5 Spatial patterns of habitat loss within salt marshes

Across the SLR scenarios, upland transition zone habitats would become suitable for BSSP with increasing inundation depth and frequency, while low elevation foraging areas were forecasted to be submerged (Figure 4). SCB salt marshes face habitat migration restrictions, such as highways, roads, and adjacent development (Figure 4), so landward movement was assumed to be minimal. Habitat loss would occur first in low areas (e.g., open water, bayward edge, and tidal creeks; Figure 4). For example, suitable habitat loss would begin near the bayward edge at Sweetwater. But habitat loss is not necessarily a landward progression. At Carpinteria, suitable breeding habitat loss would begin in the center of the salt marsh, which contains a low mudflat (Figure 4). Regardless of the progression, all currently suitable habitat areas were predicted to be inundated by SLR through the next century.

## 4 DISCUSSION

Our models suggest that under all projected SLR scenarios, and without adaptation by BSSP or accommodation by humans, near complete loss of BSSP habitat is likely throughout the SCB under high SLR scenarios. Carpinteria, currently the smallest study site, could support the last remaining BSSP population within fully tidal basins due to its relatively high‐elevation marsh.

Our results are consistent with projected declines in other mid to high salt marsh species. Seaside sparrow habitat in Georgia is expected to decline between 2025 and 2050 (Hunter et al., 2016). Under high SLR scenarios, two high‐elevation salt marsh birds, the Common Yellowthroat (*Geothlypis trichas*) and Marsh Wren (*Cistohorus palustris*), will likely become extirpated from the SFBE salt marsh within a century (Veloz et al., 2013). Small mammals, such as the salt marsh harvest mouse (*Reithrodontomys raviventris*), could be extirpated from areas currently dominated by pickleweed as sea‐levels rise and that habitat disappears (Shellhammer, 1989; Swanson et al., 2014). High to moderate SLR, coupled with low sediment supply and insufficient area for shoreward retreat, could reduce habitat for many species besides BSSP.

BSSP might not readily disperse to better sites as suitable habitat is lost. Heavy industrialization and urbanization of the landscapes of southern California might further reduce BSSP dispersal by limiting connectivity between habitats. In a 1995–1997 study, BSSP were shown to have high site fidelity; all monitored BSSP stayed within their current salt marsh (Powell & Collier, 1998). Furthermore, in the following year, 45.5% of banded male BSSPs in that same site occupied the same territory that they occupied when they were originally banded, highlighting their site fidelity. Reduced dispersal will make restoration more difficult if local populations are extirpated.

BSSP extirpation could occur before all habitats are submerged. For example, salt marshes smaller than 10 ha have been shown not to support BSSP breeding populations (Powell & Collier, 1998; Zembal et al., 1988). As habitat shrinks in area due to increasing inundation, it may also decline in quality, which might lead to breeding failure before all habitats are lost. Based on this threshold, extirpations could occur at Carpinteria and Seal Beach under a moderate SLR scenario by 2100 and 2040. Thus, a patchy distribution of marginal breeding habitat might preclude nesting well before our model predicts full breeding habitat loss.

Habitat change also depends on the extent to which that upland habitat will convert to salt marsh. Historically, this would have been a normal consequence of SLR. However, as the SCB has become more urbanized, BSSP are closer to the urban edge where they tend to do poorly. Perched BSSP react to pedestrians at distances between 47 and 63 m in southern California sites; thus, increased SLR may increase disturbance rates (Fernandez‐Juricic, Zahn, Parker, & Stankowich, 2009). Furthermore, increasing proximity to upland habitats could increase the frequency of interactions with upland predators such as red fox (*Vulpes fulva*) and raccoons (*Procyon lotor)*, species that have been detected on the edge of Carpinteria Salt Marsh (Zembal et al., 2015). Common raven (*Corvus corax*) and American crow (*Corvus brachyrynchus*) are known nest predators of several threatened and endangered species in California (Liebezeit & George, 2002), and these impacts could also increase if BSSP habitat concentrates near uplands.

Future marsh elevation and associated habitat change depend on the extent that sediment supply will make up for SLR. Large storm events in the SCB have been known to rapidly increase elevations in mudflats and low marsh zones. For example, in Tijuana, high sedimentation rates during storms have led to an increase in elevation, and low to high marsh zone habitat conversion (Ward, Callaway, & Zedler, 2003). The same is true of Mugu, where low elevation areas have been repeatedly filled with sediment during storm episodes (Onuf, 1987). The potential for extreme sedimentation and transgression is different for each of these sites. For example, although catastrophic sedimentation from the rugged Santa Ynez Mountain watersheds have buried sections of Carpinteria under 20 cm of inorganic sediments, urban development has eliminated most of the upper marsh (Callaway, Jones, Ferren, & Parikh, 1990) and has altered connectivity to freshwater sources through concrete channelization (Sadro et al., 2007). Because sediment availability is dependent on infrequent storm events that are difficult to predict (Warrick & Farnsworth, 2009), future management of sediment supply and adjacent land use will play an important role in current habitat stability. Seal Beach provides a testing ground for managing BSSP through habitat restoration and increasing tidal marsh elevation by adding dredge spoils. At a 10 ha test site, dredge materials were applied to increase elevation suitable for cordgrass (*Spartina foliosa*), however, elevations and substrate may be more suitable for pickleweed habitat in the near future.

Our analyses suggest that the recent increase in BSSP counts in the SCB (Zembal et al., 2015) will likely reverse in the near future. Even before Pacific Coast salt marshes are completely submerged in 2110 (Thorne et al., 2018), our modeling predicts that there will be no suitable habitat for BSSPs under a high SLR scenario. Although habitat suitability could temporarily increase in two of the six salt marshes we studied under low SLR scenarios, local extirpations may occur. These losses could possibly be ameliorated with management intervention, restoration, and increasing transgression upland refugia habitat.

## CONFLICT OF INTEREST

The authors have no conflict of interest to declare.

## AUTHORS CONTRIBUTION

JR conceived the study. JR and KB did the species distribution modeling, modeling of occurrences, acquired/interpreted the sediment core date data, and drafted the work. KL, RH, TS, MH, RP, and JC conceived, designed and conducted the bird surveys, habitat delineation, and revised the work critically for important intellectual content. KT and KB conducted the WARMER modeling, and revised the work critically for important intellectual content. JT, KT, RA, and GM conceived/designed the geomorphic modeling study, acquired the data for warmer modeling, and revised the work critically for important intellectual content.

## DATA ACCESSIBILITY

All data needed to evaluate the conclusions in the paper are present in the paper. Additional data related to this paper may be requested from the authors. All raw data are archived at the U.S. Geological Survey Science Base Catalog (http://www.sciencebase.gov/catalog/; https://doi.org/10.5066/F7F47M95, https://doi.org/10.5066/F7RX99V3, and https://doi.org/10.5066/F70Z728B). Any use of trade, product, or firm names in this publication is for descriptive purposes only and does not imply endorsement by the U.S. Government.
